# Supplementary material for: Sex Differences among Overweight/Obese Kidney Transplant Recipients Requiring Oxygen Support Amid the COVID-19 Pandemic
Source: Medicina (Kaunas). 2023 Aug 27;59(9):1555. doi: 10.3390/medicina59091555 (PMC10535294; doi:10.3390/medicina59091555)
Supplement: Supplementary file 1 [file medicina-59-01555-s001.zip › medicina-2535547-supplementary.pdf]

Supplementary file

Table S1. Clinical and epidemiological characteristics, laboratory data, and outcomes of male and female kidney transplant recipients.

**Variables**	**Male (n=160, 56.3%)**	**Female (n=124, 43.7%)**	**TOTAL (n=284, 100%)**	**Univariate Analysis OR (95% CI)**
Age (years)	53.2 ± 12.4	51.6 ± 11.9	52.5 ± 12.2	0.99 (0.97-1.01, p=0.28)
Race (n, %)				
White	96 (60.0)	77 (62.1)	173 (60.9)	1.09 (0.68-1.77, p=0.72)
Black/brown	64 (40.0)	47 (37.9)	111 (39.1)	
Transplant time (months)	94.7 [33.8;142.3]	91.4 [32.3;132.0]	73.5 [33.0;142.3]	1.00 (0.99-1.00, p=0.70)
Donor type (n, %)				
Live	50 (31.3)	30 (24.2)	80 (28.2)	0.70 (0.41-1.19, p=0.19)
Deceased	110 (68.7)	94 (75.8)	204 (71.8)	
Overweight (n, %)	64 (40.0)	56 (45.2)	120 (42.3)	1.24 (0.77-1.99, p=0.38)
Obesity I (n, %)	27 (16.9)	23 (18.5)	50 (17.6)	1.12 (0.61-2.07, p=0.71)
Obesity II (n, %)	2 (1.3)	7 (5.6)	9 (3.2)	4.73 (0.96-23.17, p=0.06)
Obesity III (n, %)	4 (2.5)	2 (1.6)	6 (2.1)	0.64 (0.12-3.55, p=0.61)
Hypertension (n, %)	130 (81.3)	84 (67.7)	214 (75.4)	0.49 (0.28-0.84, **p=0.01**)
Diabetes mellitus (n, %)	60 (37.5)	52 (41.9)	112 (39.4)	1.20 (0.75-1.94, p=0.45)
COPD (n, %)	5 (3.1)	4 (3.2)	9 (3.2)	1.03 (0.27-3.93, p=0.96)
Heart disease (n, %)	20 (12.5)	12 (9.7)	32 (11.3)	0.80 (0.35-1.60, p=0.46)
Neoplasia (n, %)	14 (8.8)	7 (5.6)	21 (7.4)	0.62 (0.24-1.60, p=0.33)
Liver disease (n, %)	7 (4.4)	2 (1.6)	9 (3.2)	0.36 (0.07-1.76, p=0.21)
Autoimmune disease (n, %)	1 (0.1)	7 (5.6)	8 (2.8)	9.51(1.16-78.37, **p=0.04**)
Smoking (n, %)	35 (21.9)	24 (19.4)	59 (20.8)	0.85 (0.46-1.54, p=0.59)
**Laboratory data**				
Basal eGFR	50.0 ± 24.6	47.2 ± 23.3	48.7 ± 24.0	1.00 (0.99-1.01, p=0.33)
Admission eGFR	37.8 ± 21.1	36.1 ± 22.9	37.1 ± 21.9	1.00 (0.99-1.01, p=0.52)
Previous glucose (mg/dL)	119.4 ± 56.7	121.4 ± 77.6	120.3 ± 66.5	1.00 (1.00-1.00, p=0.80)
Admission glucose (mg/dL)	180.9 ± 107.3	166.3 ± 101.0	174.8 ± 104.5	1.00 (1.00-1.00, p=0.45)
Previous Hb1Ac (%)	6.8 ± 2.0	7.0 ± 2.0	6.9 ± 2.0	1.04 (0.91-1.19, p=0.57)
CRP (mg/dL)	9.0 [1.9;13.6]	8.0 [2.2;11.0]	5.7 [2.0;12.7]	0.99 (0.96-1.02, p=0.40)
LDH (U/L)	339.7 [236.5;402.5]	320.2 [209.3;377.5]	288.0 [220.0;395.0]	1.00 (1.00-1.00, p=0.43)
Lymphocytes (mm^3)	901.0 [460.0;1094.0]	1016.3 [523.5;1298.5]	750.5 [497.0;1202.5]	1.00 (1.00-1.00, p=0.25)
D-dimer (µg/L)	2.3 [0.6;1.9]	2.4 [0.6;2.5]	1.2 [0.6;2.3]	1.01 (0.95-1.09, p=0.73)

AST (U/L)	37.3 [20.0;42.3]	40.7 [21.0;40.0]	28.0 [21.0;41.0]	1.00 (1.00-1.01, p=0.51)
ALT (U/L)	30.3 [15.0;36.0]	31.1 [14.0;27.0]	21.0 [15.0;32.0]	1.00 (0.99-1.01, p=0.89)
Sodium (mEq/L)	135.4 ± 4.4	134.5 ± 5.7	135.0 ± 5.1	0.97 (0.92-1.02, p=0.18)
**Outcomes**				
Death (n, %)	52 (32.5)	32 (25.8)	84 (29.6)	0.72 (0.43-1.22, p=0.22)
ICU (n, %)	76 (47.5)	58 (46.8)	134 (47.2)	0.97 (0.61-1.55, p=0.90)
O_2 (n, %)	82 (51.3)	71 (57.3)	153 (53.9)	1.27 (0.80-2.04, p=0.31)
IMV (n, %)	61 (38.1)	36 (29.0)	97 (34.2)	0.66 (0.40-1.10, p=0.11)
AKI (n, %)	91 (56.9)	74 (59.7)	165 (58.1)	1.12 (0.70-1.81, p=0.64)
Stage 1	17 (10.6)	20 (16.1)	37 (13.0)	1.62 (0.81-3.24, p=0.17)
Stage 2	8 (5.0)	6 (4.8)	14 (4.9)	0.97 (0.33-2.86, p=0.95)
Stage 3	66 (41.3)	48 (38.7)	114 (40.1)	0.90 (0.56-1.45, p=0.67)
HD (n, %)	64 (40.0)	41 (33.1)	105 (37.0)	0.74 (0.45-1.21, p=0.23)

BMI: body mass index in kg/m^2; COPD: chronic obstructive pulmonary disease; eGFR: estimated glomerular rate, in mL/min/1.73 m^2; Hb1Ac: glycated hemoglobin; CRP: C-reactive protein; LDH: lactate dehydrogenase; AST: aspartate aminotransferase; ALT: alanine aminotransferase; ICU: intensive care unit; O_2: use of supplemental oxygen; IMV: invasive mechanical ventilation; AKI: acute kidney injury; HD: hemodialysis. All variables are means ± SD, except the variables transplant time, CRP, LDH, lymphocytes, D-dimer, AST, and ALT, which are medians and IQR. OR: odds ratio. 95% CI: 95% confidence interval.

Tables S2-S3 denote univariate and multivariate analyzes and linear regression models using BMI as a continuous variable.

Table S2. Clinical and epidemiological characteristics, laboratory data, and outcomes of male kidney transplant recipients (n=160) using BMI as a continuous variable.

Variables	Univariate Analysis OR (95% CI)	Multivariate Analysis OR (95% CI)
Age (years)	0.00 (-0.059-0.064, p=0.94)	
Race (n, %)		
White	0.23 (-1.324-1.785, p=0.77)	
Black/brown		
Transplant time (months)	0.01 (-0.005-0.016, p=0.33)	
Donor type (n, %)		
Live	1.81 (0.188-3.426, **p=0.03**)	1.98 (0.376-3.582, **p=0.02**)
Deceased		
Hypertension (n, %)	2.04 (0.115-3.966, **p=0.04**)	2.25 (0.350-4.157, **p=0.02**)
Diabetes mellitus (n, %)	0.36 (-1.210-1.936, p=0.65)	
COPD (n, %)	-1.07 (-5.447-3.304, p=0.63)	
Heart disease (n, %)	0.30 (-2.004-2.602, p=0.80)	
Neoplasia (n, %)	-0.46 (-3.155-2.235, p=0.74)	
Liver disease (n, %)	-0.83 (-4.553-2.892, p=0.66)	
Autoimmune disease (n, %)	-4.02 (-13.666-5.627, p=0.42)	
Smoking (n, %)	-0.20 (-2.116-1.724, p=0.84)	
**Laboratory data**		
Basal eGFR	0.01 (-0.018-0.044, p=0.42)	
Admission eGFR	0.03 (-0.007-0.065, p=0.12)	
Previous glucose (mg/dL)	0.02 (0.001-0.028, **p=0.03**)	0.01 (-0.009-0.026, p=0.33)
Admission glucose (mg/dL)	0.01 (-0.003-0.020, p=0.14)	
Previous Hb1Ac (%)	0.14 (-0.292-0.567, p=0.53)	
CRP (mg/dL)	0.02 (-0.078-0.116, p=0.70)	
LDH (U/L)	0.01 (-0.001-0.010, p=0.09)	0.00 (-0.001-0.010, p=0.10)
Lymphocytes (mm^3)	0.00 (-0.001-0.001, p=0.56)	
D-dimer (μg/L)	0.01 (-0.201-0.229, p=0.90)	
AST (U/L)	0.02 (-0.011-0.046, p=0.23)	
ALT (U/L)	0.01 (-0.015-0.042, p=0.35)	
Sodium (mEq/L)	0.07 (-0.125-0.255, p=0.50)	
**Outcomes**		
Death (n, %)	0.66 (-0.963-2.284, p=0.42)	
ICU (n, %)	0.34 (-1.181-1.869, p=0.66)	
O_2 (n, %)	0.26 (-1.266-1.781, p=0.74)	
IMV (n, %)	0.99 (-0.572-2.549, p=0.21)	
AKI (n, %)	-1.21 (-2.732-0.321, p=0.12)	
Stage 1	-2.29 (-4.738-0.153, p=0.07)	-2.53 (-4.954--0.112, **p=0.04**)
Stage 2	-3.93 (-7.374--0.493, **p=0.02**)	-4.22 (-7.632--0.800, **p=0.02**)
Stage 3	0.45 (-1.097-1.995, p=0.57)	
HD (n, %)	0.57 (-0.980-2.125, p=0.47)	

BMI: body mass index in kg/m^2; COPD: chronic obstructive pulmonary disease; eGFR: estimated glomerular rate, in mL/min/1.73 m^2; Hb1Ac: glycated hemoglobin; CRP: C-reactive protein; LDH: lactate dehydrogenase; AST: aspartate aminotransferase; ALT: alanine aminotransferase; ICU: intensive care unit; O_2: use of supplemental oxygen; IMV: invasive mechanical ventilation; AKI: acute kidney injury; HD: hemodialysis. OR: odds ratio. 95% CI: 95% confidence interval.

Table S3. Clinical and epidemiological characteristics, laboratory data, and outcomes of female kidney transplant recipients (n=124) using BMI as a continuous variable.

Variables	Univariate Analysis OR (95% CI)	Multivariate Analysis OR (95% CI)
Age (years)	0.05 (-0.020-0.126, p=0.15)	
Race (n, %)		
White	1.38 (-0.405-3.159, p=0.13)	
Black/brown		
Transplant time (months)	-0.01 (-0.018-0.007, p=0.40)	
Donor type (n, %)		
Live	-0.35 (-2.391-1.684, p=0.73)	
Deceased		
Hypertension (n, %)	0.65 (-1.213-2.514, p=0.49)	
Diabetes mellitus (n, %)	1.19 (-0.566-2.946, p=0.18)	
COPD (n, %)	-2.28 (-7.201-2.645, p=0.36)	
Heart disease (n, %)	2.44 (-0.478-5.361, p=0.10)	
Neoplasia (n, %)	0.58 (-3.198-4.364, p=0.76)	
Liver disease (n, %)	-0.47 (-7.393-6.465, p=0.89)	
Autoimmune disease (n, %)	0.15 (-3.632-3.932, p=0.94)	
Smoking (n, %)	0.19 (-2.120-2.491, p=0.87)	
**Laboratory data**		
Basal eGFR	0.02 (-0.016-0.059, p=0.25)	
Admission eGFR	0.02 (-0.022-0.055, p=0.39)	
Previous glucose (mg/dL)	0.01 (-0.006-0.017, p=0.36)	
Admission glucose (mg/dL)	0.01 (-0.006-0.023, p=0.24)	
Previous Hb1Ac (%)	0.82 (0.287-1.352, **p=0.003**)	
CRP (mg/dL)	-0.01 (-0.119-0.100, p=0.86)	
LDH (U/L)	0.00 (-0.003-0.008, p=0.34)	
Lymphocytes (mm^3)	0.00 (-0.001-0.002, p=0.70)	
D-dimer (µg/L)	-0.24 (-0.535-0.054, p=0.11)	
AST (U/L)	0.01 (-0.010-0.033, p=0.29)	
ALT (U/L)	0.01 (-0.008-0.026, p=0.31)	
Sodium (mEq/L)	0.10 (-0.063-0.258, p=0.23)	
**Outcomes**		
Death (n, %)	-0.27 (-2.262-1.726, p=0.79)	
ICU (n, %)	1.46 (-0.272-3.187, p=0.10)	0.02 (-2.069-2.106, p=0.99)
O_2 (n, %)	2.51 (0.801-4.214, **p=0.004**)	2.50 (0.391-4.602, **p=0.02**)
IMV (n, %)	1.10 (-0.814-3.012, p=0.26)	
AKI (n, %)	-0.13 (-1.913-1.646, p=0.88)	
Stage 1	0.76 (-1.614-3.125, p=0.53)	
Stage 2	0.92 (-3.149-4.980, p=0.66)	
Stage 3	-0.74 (-2.531-1.043, p=0.41)	
HD (n, %)	0.11 (-1.745-1.965, p=0.91)	

BMI: body mass index in kg/m^2; COPD: chronic obstructive pulmonary disease; eGFR: estimated glomerular rate, in mL/min/1.73 m^2; Hb1Ac: glycated hemoglobin; CRP: C-reactive protein; LDH: lactate dehydrogenase; AST: aspartate aminotransferase; ALT: alanine aminotransferase; ICU: intensive care unit; O_2: use of supplemental oxygen; IMV: invasive mechanical ventilation; AKI: acute kidney injury; HD: hemodialysis. OR: odds ratio. 95% CI: 95% confidence interval.
